# Cues Paired with either Rapid or Slower Self-Administered Cocaine Injections Acquire Similar Conditioned Rewarding Properties

**DOI:** 10.1371/journal.pone.0026481

**Published:** 2011-10-19

**Authors:** Anne-Noël Samaha, Ellie-Anna Minogianis, Walid Nachar

**Affiliations:** 1 Department of Pharmacology, Faculty of Medicine, Université de Montréal, Montréal, Québec, Canada; 2 CNS Research Group, Faculty of Medicine, Université de Montréal, Montréal, Québec, Canada; Centre for Addiction and Mental Health, Canada

## Abstract

The faster drugs of abuse reach the brain, the more addictive they can be. It is not known why this is. Environmental stimuli associated with drugs can promote the development and persistence of addiction by invigorating and precipitating drug-seeking behaviour. We determined, therefore, whether cues associated with the self-administration of rapidly delivered cocaine (injected intravenously over 5 versus 90 seconds) would acquire greater conditioned rewarding properties, as assessed by the performance of an operant response reinforced solely by the cues. Rats nose-poked for intravenous cocaine infusions delivered either over 5 or 90 seconds. Discrete visual cues accompanied each infusion. The rats could then press a lever to obtain the cues—now a conditioned reward—or an inactive lever. Rats in both the 5- and 90-second groups pressed more on the active versus inactive lever following extensive (24 sessions) but not following limited (3 sessions) self-administration training. There were no group differences in this behaviour. Following withdrawal from cocaine self-administration, lever discrimination progressively abated in both groups and was lost by withdrawal day 30. However, the rewarding properties of the cues were not “forgotten” because on withdrawal days 32–33, amphetamine selectively enhanced active-lever pressing, and did so to a similar extent in both groups. Thus, cues paired with rapid or slower cocaine delivery acquire similar conditioned rewarding properties. We conclude, therefore, that the rapid delivery of cocaine to the brain promotes addiction by mechanisms that might not involve a greater ability of drug cues to control behaviour.

## Introduction

When drugs reach the brain quickly the probability and severity of addiction are increased. For example, smoking or intravenously (i.v.) injecting cocaine are the two fastest routes for getting the drug to the brain and these routes are associated with greater self-reports of loss of control over drug use, greater difficulty in reducing or stopping cocaine use and an increased likelihood of developing addiction [1]–[7]. In laboratory animals, increasing the speed of intravenous drug delivery promotes the development of psychomotor sensitization to cocaine [8], [9] and nicotine [10], increases the motivation to self-administer cocaine over repeated test sessions [11] (although not acutely [11], [12]) and leads to both greater cocaine intake under extended-access conditions and more persistent vulnerability to drug-primed reinstatement of extinguished cocaine-seeking behaviour [13]. With respect to the effects of drugs on the brain, ‘how quickly’ appears to be just as decisive as ‘how much’ [14]. When drugs like cocaine or nicotine are delivered to the brain rapidly, they lead to greater changes in cellular activity in mesocorticolimbic structures [9], [10], [15], greater and more immediate increases in heat-producing, metabolic activity in the ventral tegmental area and nucleus accumbens [14], and more immediate increases in dopamine transporter blockade [9] and extracellular dopamine levels [16] in the striatum.

It has been proposed that rapidly delivered drugs are more addictive because they can lead to more intense and more immediate subjective pleasurable effects [17]–[19], because they are more acutely reinforcing [20]–[23], and/or because they promote sensitization-related changes in the brain that promote excessive incentive motivation for drugs [8]–[10], [24]. In addition to these drug effects, stimuli that have come to be associated with drug use can also contribute in powerful ways to the initiation and persistence of drug addiction [25]. For a given drug user, there are specific objects, people, sounds, places and sensations that consistently precede the onset of drug effects. In cocaine-experienced individuals, for example, it has been proposed that such exteroceptive and interoceptive stimuli cause more direct and more immediate reward signalling than the drug itself [26]. In addicts, such stimuli are known to elicit attention and approach [27]–[29], become excessively wanted (in “needle freaks” for example, who compulsively inject with a drug-free needle [30]) and invoke motivational states that can support compulsive drug seeking and induce relapse during abstinence [25], [31]. Similarly, in laboratory animals, drug-associated stimuli elicit approach [32], precipitate or energize drug-seeking behaviour [33], [34] and instigate reinstatement of previously extinguished drug-seeking behaviour [35]–[37].

Reward-associated cues can act as “response activators”, triggering and energizing reward-seeking behaviours [38]–[40]. They can also support the spontaneous learning of novel instrumental actions, i.e. they can act as conditioned reinforcers [41]. Indeed, environmental cues paired with self-administered drugs such as cocaine or heroin [42], [43] can support the rapid learning of novel operant behaviours, in a manner that is persistent and strictly dependent upon the associative strength between the cue and the reward. Environmental cues acquire greater associative strength and do so sooner when the time interval separating presentation of the cues and the unconditioned effects of the reward is short [44]. A subject smoking or intravenously injecting cocaine will experience the unconditioned central nervous system actions of the drug more immediately than a subject using a slower route of administration (e.g., the intranasal route). Consequently, any cues that precede cocaine smoking or i.v. injection might be expected to acquire greater associative strength, and do so sooner than stimuli associated with the slower delivery of the drug to the brain. In considering this, we asked the following question: Do environmental stimuli associated with rapid versus more sustained cocaine delivery acquire greater conditioned rewarding properties, and do so sooner? To address this question, we allowed rats to self-administer intravenous cocaine injections delivered either over 5 or 90 seconds (s). Discrete visual cues accompanied each cocaine injection, such that these became drug cues. We then assessed the conditioned rewarding properties of these cues by examining the acquisition and persistence of operant responding reinforced solely by the cues. Finally, we assessed amphetamine-induced potentiation of operant responding for the cues, as amphetamine [45]—but not cocaine [46]—is known to enhance the incentive motivational properties of Pavlovian reward cues.

## Materials and Methods

### Ethics Statement

The experimental procedures were performed in accordance with the principles outlined by the Canadian Council on Animal Care. The Committee on the Ethics of Animal Experiments of the Université de Montréal approved all experiments (Protocol Number: 10-106). The experimenters made all reasonable efforts to avoid or minimize animal suffering.

### Subjects

Male *Wistar* rats (Charles River Laboratories, St-Constant, Quebec; weighing 225–250 g upon arrival) were individually housed under a 12-h reverse light/dark cycle (lights off at 8:00 AM). Testing was conducted during the dark phase of the animals' circadian cycle. Unless indicated otherwise, water was available *ad libitum*. Unless indicated otherwise, food was restricted to 5 standard rodent food pellets (23–25 g) per day, given 1–2 h after daily testing.

### Drugs

Cocaine hydrochloride (Medisca Pharmaceutique Inc., St-Laurent, Quebec) was dissolved in 0.9% saline. The concentration of cocaine solutions was adjusted every 3–4 days according to the average body weight of the animals. D-amphetamine sulphate (AMPH; Sigma-Aldrich, Dorset, UK) was dissolved in 0.9% saline, and given subcutaneously (SC) in a volume of 1 ml/kg.

### Apparatus

Rats were tested in standard operant cages (31.8×25.4×26.7 cm; Med Associates Inc, St-Albans, VT) placed in a testing room different from their housing area. One wall of each operant cage was equipped with two 4 cm-wide levers. The two levers were 12 cm apart, and located 8 cm above the grid floor. A white cue light was located above each lever, and a white house light was located at the top of the opposite wall. A sonalert (tone-generator) was located at the top right of the house light, and it was calibrated to produce a 2900-Hz, 85-dB tone. A food magazine placed in a recessed port was located in between the two levers. Infrared beams detected head entries (nose pokes) into this port. Experiments 1 and 2 were carried out in twenty-four cages containing one retractable lever located on the left of the food magazine and one fixed lever located on the right of the food magazine. Experiment 1b was carried out in six cages containing two retractable levers, located on each side of the food magazine. Absorbent wood chips were placed on the floor of each operant cage. The floor was covered with a metal grid. Each testing cage was placed in a larger sound-light attenuating cabinet equipped with a ventilation fan that also masked external noise. Each chamber was equipped with a PHM-100 infusion pump (Med Associates). A 10-ml, cocaine-containing syringe was placed upon each pump and attached to a liquid swivel (Lomir Biomedical Inc., Notre-Dame-de-l'Île-Perrot, Quebec) via Tygon tubing. The swivel was mounted on a counter-balanced arm that allowed free movement of the animal. The animals' catheters were connected to the swivel with Tygon tubing shielded with a metal spring. The operant chambers were interfaced to software (Med Associates Med-PC version IV).

### Food Training

To facilitate the acquisition of cocaine self-administration behaviour, rats underwent operant training using a food reward. Rats were restricted to 3 large food pellets (∼15 g) for three days prior the first operant food training day. On each of two sessions, rats were placed in the operant cages, and learned to nose poke for banana-flavoured food pellets (45 mg, grain-based, Dustless Precision Pellets; VWR, Montreal, Quebec) under a continuous schedule of reinforcement (fixed ratio 1; FR 1). No discrete cues (lights or tones) were associated with the delivery of food save for the sounds made by the pellet dispenser and the delivery of each pellet into the food receptacle. Sessions ended after 30 minutes or 100 responses. Upon completion of each session, rats were returned to their home cages and given 2 large food pellets. Animals that did not nose-poke at least 25 times during the second session were retrained overnight. Overnight sessions ended after 300 responses or were manually interrupted at 8:00 AM the next morning.

### Jugular Catheterization

Two days following food training, each rat was implanted with a catheter into the external jugular vein under 2% isoflurane anaesthesia. Catheters were homemade and consisted of a cannula attached to a 12.5-cm length of silastic tubing (ID = 0.51 mm, OD = 0.94 mm) and fixed to nylon mesh with dental cement. A silicone bubble was placed 3.3 cm from the bevelled tip of the silastic tubing and another was placed 1.7 cm above the first bubble. After inserting the bevelled tip of the catheter into the jugular vein, one of the silicone bubbles was used to tie the catheter to the vein, while the other bubble was used to anchor the catheter to the chest muscle. The other end of the catheter was fed subcutaneously under the front paw to protrude from the scapular region. At the time of surgery, rats were given an intra-muscular injection of 0.3 ml of a penicillin solution (Derapen, 300 mg/ml; CDMV, Saint-Hyacinthe, Quebec) and a SC injection of 5 mg/kg Carprofen (Rimadyl; 50 mg/ml; CDMV).

#### Experiment 1

**Figure 1:**
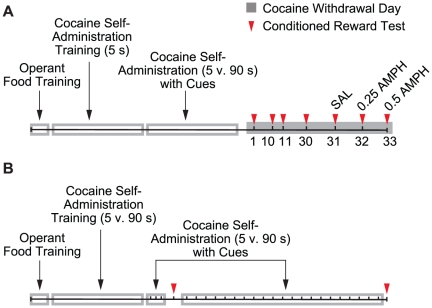
Timeline of behavioural training and testing. In Experiment 1 (panel A) we examined the effects of the speed of intravenous cocaine delivery on the acquisition, persistence and AMPH-induced potentiation of operant responding for a cocaine-paired cue. In Experiment 2 (panel B) we determined whether cues paired with rapid self-administered cocaine infusions would acquire conditioned reinforcing properties sooner than cues paired with slower cocaine infusions. s, seconds; SAL, saline; AMPH, amphetamine.

The objective here was to determine the effects of the speed of intravenous cocaine delivery on the acquisition, persistence and AMPH-induced potentiation of operant responding for a cocaine-paired cue. The sequence of experimental events is illustrated in Figure 1A.

*Cocaine self-administration training without drug cues*. Two to seven days following jugular catheterization, all rats were trained to nose-poke for cocaine infusions (0.5 mg/kg/infusion; weight of the salt) delivered intravenously over 5 s under an FR 1 schedule of reinforcement. Each infusion was followed by a 20-s time out period during which additional nose-pokes were recorded but did not deliver cocaine. Before each session, catheters were flushed with 0.1 ml sterile saline. On alternate sessions, catheters were flushed with 0.1 ml of a saline solution containing 0.2 mg/ml heparin (Sigma-Aldrich, Oakville, Ontario) and 2 mg/ml enrofloxacine (Baytril, 100 mg/ml; CDMV), at the end of the session. At the beginning of each session, rats were attached to the infusion apparatus and placed in the operant cages. Sessions were conducted once a day and lasted for 90 minutes. The left lever remained retracted at all times and no experimenter-programmed cues were explicitly paired with cocaine during training. Once rats met minimum training criteria (≥5 infusions/session, taken at regular intervals throughout the session according to visual inspection of the cumulative-response record), the ratio was increased to FR 2, and the time out period was increased to 45 s, then 65 s, and finally 85 s. This is because we ultimately wished to study the effects of 5- versus 90-s cocaine infusions, and an 85-s time out period ensured that regardless of infusion speed, all rats could take only one injection every 90 s. Rats remained on an FR 2–85 s time out schedule for five consecutive days before beginning cocaine self-administration with cues.

*Cocaine self-administration with drug cues.* Animals were divided into two groups where they could self-administer cocaine infusions delivered over 5 or 90 s. Group assignment was made such that the mean number of infusions obtained during the last 2 days of training was equivalent in the two groups. Five- or 90-s infusions were delivered using syringe pump motors (model R-DE; Med Associates) capable of delivering 150 µl over 5 s (3.33 rpm) or 80 µl over 90 s (0.1 rpm), respectively. Rats could nose-poke for cocaine under an FR 2 schedule. Each infusion was followed by an 85-s time out period for the 5-s group and no time out for the 90-s group. During the actual infusion and time out period (if applicable), the stimulus light above the retracted left lever was lit. Thus, upon each drug infusion, the drug cue (i.e., illumination of the stimulus light) was presented for 90 s in both groups. To hold the number of infusions (and thus the number of cue-cocaine pairings) constant, sessions initially ended after 10 infusions or 3 hours. Once a rat obtained the required number of infusions over two consecutive sessions, the infusion criterion (IC) was increased to 15, 20, 25 and finally 30 infusions/session. Rats remained at IC 30 for 5 sessions before conditioned reward testing. On occasion, some rats did not take the required number of infusions prior to the 3-h time limit. For this reason, there was some variability in the total number of cue-cocaine pairings in each of the experimental groups (see Results).

*Operant responding for conditioned reward.* The conditioned rewarding properties of the cocaine cue were measured by assessing the acquisition of a new lever-pressing response reinforced solely by the cue. Rats were placed in the operant chambers and tethered to the intravenous drug infusion lines, with the drug pumps turned off. The left lever was inserted into the chamber for the very first time. Rats could press this lever (Conditioned Reward lever; CR) to obtain 5-s presentations of the cocaine cue (i.e., illumination of the stimulus light above the left lever—now a conditioned reward) according to a random-ratio 2 schedule. Rats could also press an inactive lever (Non-Conditioned Reward lever; NCR) that produced no consequences. No cocaine was delivered. Sessions lasted for 40 min, based on [42]. Conditioned reward tests were given 1, 10–11 and 30 days after the last cocaine self-administration session with cues. Three additional conditioned reward tests were given 31, 32 and 33 days following cocaine withdrawal. Five minutes before each of these last three tests, rats were injected SC with saline (first test), 0.25 (second test) or 0.5 (third test) mg/kg AMPH. The order of the tested doses was set to minimize the likelihood of carry-over and/or sensitization effects.

#### Experiment 1b

On the operant responding for conditioned reward tests in Experiment 1 above and Experiment 2 below, the CR lever was novel and the NCR lever was familiar to the rats. This is because the NCR was non-retractable and was present during previous cocaine self-administration sessions. However, the CR was retractable and inserted into the operant chamber for the first time on conditioned reward testing. The objective here was to determine whether CR vs. NCR lever discrimination would be apparent on a test for conditioned reward when both levers are novel.

*Pavlovian conditioning and operant responding for conditioned reward.* Water-restricted rats (2 h/day) were placed individually in standard operant chambers containing two retractable levers. Both levers remained retracted during this phase. The rats were trained to associate the delivery of 0.1 ml tap water (the unconditioned stimulus; UCS) into a receptacle with presentations of a light/tone stimulus (the conditioned stimulus; CS), as in [47]. CS-UCS training occurred once a day for 10 days. Following conditioning, rats were placed individually in the operant chambers with both levers present for the first time. On this session, pressing on one of the two levers (CR lever) produced 5-s presentations of the CS (now a conditioned reward) according to a random-ratio 2 schedule, and pressing on the other lever (NCR lever) produced no consequences. No water was delivered and sessions lasted for 40 min.

#### Experiment 2

The objective here was to determine whether cues paired with rapid self-administered cocaine infusions would acquire conditioned reinforcing properties sooner than cues paired with slower cocaine infusions. The sequence of experimental events is illustrated in Figure 1B. Procedures were identical to those of Experiment 1 except where noted below.

*Cocaine self-administration training without drug cues.* Unlike in Experiment 1, where rats were initially trained to self-administer cocaine at the same infusion speed (i.e., 5 s) before being assigned to either the 5- or 90-s group, rats were now trained to nose-poke for cocaine (0.5 mg/kg/infusion) delivered either over 5 or 90-s under an FR 1 schedule of reinforcement, with an 85 s time out period for the 5-s group, straight from the outset. This was done in order to avoid exposing rats in the 90-s group to 5-s cocaine infusions. Once rats met acquisition criteria (≥5 infusions/session, taken at regular intervals throughout the session according to visual inspection of the cumulative-response record), they were moved to an FR 2 schedule, where they had to meet the same acquisition criteria before proceeding to cocaine self-administration with drug cues. Self-administration proceeded under an FR 2 schedule of reinforcement for the remainder of the experiment.

*Cocaine self-administration with drug cues.* In order to increase the saliency of cocaine-paired cues during this phase, each infusion was accompanied by illumination of the stimulus light above the retracted left lever (as described in Experiment 1) as well as extinction of the house light. These stimuli were presented during each cocaine infusion in both the 5- and 90-s groups, as well as during each 85-s timeout period in the 5-s group. Thus, in both groups, drug-paired cues were presented for 90 s upon each cocaine infusion. Initially, sessions ended after 10 infusions or 3 hours, for three consecutive sessions. The IC was then increased to 15 for two consecutive sessions, before being increased to 20. Rats remained at IC 20 for 21 sessions. As in Experiment 1, on occasion some rats did not satisfy the IC prior to the 3-hour time limit. Thus, there was some variability in total drug intake and total number of cue-cocaine pairings in each of the experimental groups (see Results).

*Operant responding for conditioned reward.* A first conditioned reward test was given on the day following 3 consecutive sessions at IC 10, and a second test was given 1–2 days following an additional 21 consecutive sessions at IC 20 (i.e., following a total of 24 cocaine self-administration sessions). Unlike in Experiment 1, the duration of cue presentation during these tests was shortened from 5 to 1 s. This was done in the hopes of increasing the number of active lever presses to levels previously reported [42].

### Statistics

Total drug intake and total number of cues-cocaine pairings were analyzed with unpaired *t*-tests. Number of days to reach each ratio or infusion criterion was analyzed with repeated-measures two-way ANOVA. Lever presses were analyzed with three-way ANOVA. When main effects were significant, these were further investigated using paired *t*-tests. In Experiment 1, significant lever x AMPH dose interaction effects were further analyzed using Helmert contrasts. In Experiment 2, significant lever x self-administration experience interaction effects were further analyzed using paired *t*-tests. A 2-tailed *p* value of less than 0.05 was considered significant.

## Results

### Experiment 1: Acquisition, persistence and AMPH-induced potentiation of operant responding for a cocaine-paired cue

**Figure 2:**
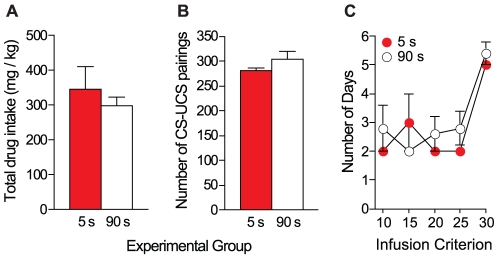
In Experiment 1, there were no differences in the amount of cocaine exposure, cocaine-cue exposure, or number of days to reach each infusion criterion between the 5- and 90-s groups. Total drug intake (panel A), number of cue-cocaine pairings (panel B) and days to reach infusion criteria (panel C). Note that total drug intake includes cocaine taken during self-administration training, when all rats self-administered cocaine injections delivered over 5 seconds. Rats were required to meet infusion criteria 10–25 for 2 days each and infusion criterion 30 for 5 days. Values are mean ± SEM. n's = 5/group. s, seconds. CS, conditioned stimulus; UCS; unconditioned stimulus.

All rats were first required to acquire cocaine self-administration behaviour, where each cocaine infusion was delivered over 5 s and followed by an 85-s time out period during which drug was unavailable. Rats were then divided into the 5- and 90-s groups where each self-administered cocaine infusion was delivered either over 5 or 90 s and now accompanied by a light cue. During the cocaine-cue self-administration phase, rats were required to meet a progressively increasing criterion of 10, 15, 20 and 25 infusions/session, for 2 consecutive days each, and then an infusion criterion of 30 for 5 days. Figure 2 shows the total amount of cocaine consumed (A), the total number of cue-cocaine pairings earned (B) and the number of days to reach each infusion criterion (C) by rats in the 5- and 90-s groups. Note that the total amount of cocaine consumed includes cocaine taken during training, when all rats self-administered cocaine injections delivered over 5 s. The two groups were exposed to a similar total quantity of cocaine [(A), *t*(8) = 0.69, *p* = 0.51] and total number of cue-cocaine pairings [(B), *t*(8) = 1.4, *p* = 0.20]. In addition, there were no group differences in the mean ± SEM number of days to meet each infusion criterion [(C), *F*(1, 8) = 0.81, *p* = 0.39].

**Figure 3:**
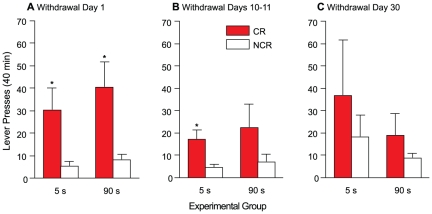
A cue paired with either rapid or slower cocaine injections acquires similar conditioned rewarding properties, and these properties abate with time. Presses on the CR and NCR levers on Day 1 (panel A), Days 10–11 (panel B) and Day 30 (panel C) of withdrawal from self-administered cocaine in the 5- and 90-s groups. Values are mean ± SEM. n's = 5/group. s, seconds; CR, conditioned reward lever; NCR, non-conditioned reward lever. *p<0.05 compared with NCR within the same group.

Figure 3 shows CR versus NCR lever presses as a function of group, one day (A), 10–11 days (B) and 30 days (C) following the last cocaine self-administration session. There was no main effect of group (*F*(1, 8) = 0.012, *p* = 0.92). There was a significant overall main effect of lever (*F*(1, 8) = 10.63, *p* = 0.012). Further investigation of the effect of lever revealed that in the 5-s group, CR lever presses were greater than NCR lever presses on Day 1 [(A) *t*(4) = 2.77, *p* = 0.049] and on Days 10–11 [(B) *t*(4) = 3.07, *p* = 0.037], but not on Day 30 [(C) *t*(4) = 1.15, *p* = 0.31] of cocaine withdrawal. In the 90-s group, CR lever presses were greater than NCR lever presses only on Day 1 of cocaine withdrawal [(A) *t*(4) = 3.30, *p* = 0.03; (B) *t*(4) = 2.00, *p* = 0.12; (C) *t*(4) = 1.26, *p* = 0.28]. This indicates that early following withdrawal from cocaine self-administration, both groups discriminated between the two levers and spontaneously acquired a new operant response, reinforced solely by the conditioned reward. However, in both groups, lever discrimination was no longer evident following more extended withdrawal from cocaine.

**Figure 4:**
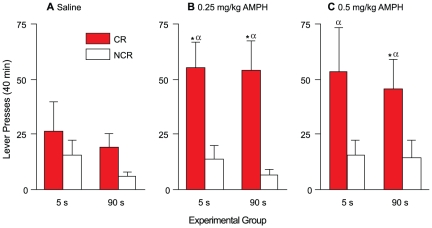
Amphetamine potentiates CR, but not NCR, lever presses in both the 5- and 90-s groups. Presses on the CR and NCR levers following an acute injection of saline (panel A), 0.25 (panel B) and 0.5 (panel C) mg/kg amphetamine. Values are mean ± SEM. n's = 5/group. s, seconds; CR, conditioned reward lever; NCR, non-conditioned reward lever; AMPH, amphetamine. *p<0.05 compared with NCR within the same group. α *p*<0.05 compared with CR under saline.

Figure 4 shows CR versus NCR lever presses as a function of group, following an injection of saline (A), 0.25 (B) and 0.5 (C) mg/kg AMPH. There was no main effect of group (*F*(1, 8) = 0.337, *p* = 0.58). There was a significant main effect of lever (*F*(1, 8) = 14.72, *p* = 0.005). Further investigation of the effect of lever revealed that in the 5-s group, CR lever presses were greater than NCR lever presses following an injection of 0.25 mg/kg AMPH [(B) *t*(4) = 3.63, *p* = 0.02], but not following saline [(A) *t*(4) = 0.70, *p* = 0.52] or an injection of 0.5 mg/kg AMPH [(C) *t*(4) = 1.95, *p* = 0.12]. For the 90-s group, CR lever presses were greater than NCR lever presses following an injection of either 0.25 mg/kg [(B) *t*(4) = 3.48, *p* = 0.025] or 0.5 mg/kg AMPH [(C) *t*(4) = 2.97, *p* = 0.041], but not following an injection of saline [(A) *t*(4) = 2.87, *p* = 0.05]. Thus, both groups showed no lever discrimination following an injection of saline, but pressed more on the CR vs. NCR lever following an acute AMPH challenge. In addition, there was a significant lever x AMPH dose interaction (*F*(1, 8) = 9.67, *p* = 0.014), indicating that the effect of AMPH on lever pressing depended on lever type. To investigate the lever x AMPH dose interaction, we collapsed the 5- and 90-s groups together and used Helmert contrasts to compare saline to the two AMPH doses and the two AMPH doses to each other. This confirmed that both 0.25 and 0.5 mg/kg AMPH increased CR lever pressing relative to saline (*F*(1, 9) = 14.293, *p* = 0.004), and that there was no difference between the two AMPH doses (*F*(1, 9) = 1.41, *p* = 0.27).

### Experiment 1b: Operant responding for conditioned reward when both response levers are novel

Figure S1 shows that when in the presence of two novel levers, rats pressed more on the lever that produced a conditioned reward (a light-tone cue previously paired with the delivery of water; CR lever) than on the lever that did not [NCR lever; Paired t-test, *t*(6) = 7.06, *p* = 0.0004]. Thus, rats discriminated between the two novel levers and spontaneously acquired a new operant response, reinforced solely by the conditioned reward.

### Experiment 2: Operant responding for cocaine-paired cues following limited versus extensive cocaine self-administration

**Figure 5:**
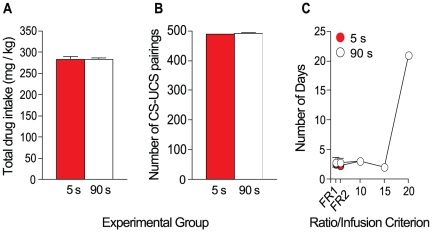
In Experiment 2, there were no differences in the amount of cocaine exposure, cocaine-cue exposure, or the number of days to reach each ratio/infusion criterion between the 5- and 90-s groups. Total drug intake (panel A), number of cue-cocaine pairings (panel B) and days to reach ratio/infusion criteria (panel C) in the 5- and 90-s groups. Rats were required to meet the FR 1 and FR 2 criteria as well as infusion criteria 10–15 for 2 days each, and to meet infusion criterion 20 for 21 days. Values are mean ± SEM. n's = 6–7/group. s, seconds. CS, conditioned stimulus; UCS; unconditioned stimulus. FR; fixed ratio.

In experiment 2, rats were trained to nose-poke for cocaine delivered either over 5 or 90 s, with an 85-s time out period for the 5-s group, first under an FR 1 schedule, and then under FR 2. Acquisition criteria were defined as taking ≥5 infusions/session, at regular intervals throughout the session. Acquisition criteria had to be met for two consecutive days under each ratio schedule. Next, rats were allowed to self-administer cocaine infusions delivered either over 5 or 90 s and accompanied by light cues. During this phase, the rats were required to meet a criterion of 10 and then 15 infusions/session, for 2 consecutive days each, and then an infusion criterion of 20 for 21 days. Figure 5 shows the total amount of cocaine consumed (A), the total number of cue-cocaine pairings earned (B) and the number of days to reach each ratio and infusion criterion (C) by rats in the 5- and 90-s groups. There was no group difference in the total quantity of cocaine consumed [(A), *t*(11) = 0.19, *p* = 0.85] or the total number of cue-cocaine pairings earned [(B), *t*(11) = 0.24, *p* = 0.82]. In addition, there were no group differences in the mean ± SEM number of days to meet each ratio and infusion criterion [(C), *F*(1, 11) = 0.58, *p* = 0.46].

**Figure 6:**
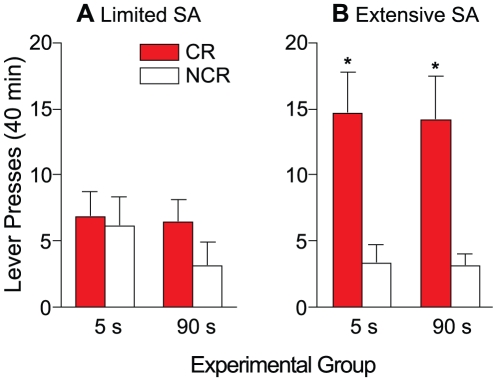
In both the 5- and 90-s groups, discrimination between the CR and NCR levers is observed following extensive (panel B) but not limited (panel A) cocaine self-administration experience. Limited self-administration experience consisted of 3 self-administration sessions. Extensive self-administration experience consisted of 24 self-administration sessions. Values are mean ± SEM. n's = 6–7/group. s, seconds; CR, conditioned reward lever; NCR, non-conditioned reward lever; SA, cocaine self-administration. *p<0.05 compared with NCR within the same group.

Figure 6 shows CR versus NCR lever presses as a function of group, following three (A) or twenty-four (B) cocaine self-administration sessions. There was no main effect of group (*F*(1, 11) = 0.28, *p* = 0.61). There was an overall main effect of lever (*F*(1, 11) = 27.8, *p* = 0.000) and a significant lever x self-administration experience interaction (*F*(1, 11) = 8.202, *p* = 0.015), indicating that lever discrimination differed as a function of self-administration experience. Further investigation of this interaction effect revealed that in both the 5- and 90-s groups, there was no difference in CR vs. NCR lever pressing following limited cocaine self-administration experience [i.e., 3 self-administration sessions; (A), 5-s group, *t*(6) = 0.18, *p* = 0.87; 90-s group, *t*(5) = 1.58, *p* = 0.18 ], but that CR lever presses were greater than NCR lever presses following extensive cocaine self-administration experience [i.e., 24 self-administration sessions; (B), 5-s group, *t*(6) = 4.168, *p* = 0.006; 90-s group, *t*(5) = 4.437, *p* = 0.007]. Thus, following extensive, but not limited self-administration experience, both groups discriminated between the two levers and spontaneously acquired a new operant response, reinforced solely by the conditioned reward.

## Discussion

To our knowledge, the present study is the first and only report examining the potential effects of the speed of cocaine delivery on the response to drug cues. Our objectives were to determine whether environmental cues paired with the rapid i.v. delivery of cocaine might be invested with greater conditioned rewarding properties, and acquire such properties sooner, than cues paired with more sustained cocaine delivery. We found that—save for a small effect on the persistence of operant responding for cocaine-paired cues (see Figure 3B)—the speed of cocaine delivery did not significantly influence the conditioned rewarding effects of drug cues. Both animals that chronically self-administered rapid (delivered i.v. over 5 s) or more sustained (90 s) cocaine injections paired with discrete visual cues spontaneously acquired a novel operant response reinforced solely by the cues, performed this response with the same vigour, showed equivalent levels of amphetamine-induced potentiation of operant responding for the cues, and required a similar number of cue-cocaine pairings to pursue the cues. Thus, discrete cues associated with the rapid entry of cocaine into the brain were no more reinforcing than cues associated with more sustained cocaine delivery, at least over the range of delivery speeds used here (5 vs. 90 s). This range of injection speeds produces different magnitudes of subjective effects [48], estimates the different rates of rise of plasma cocaine levels when cocaine is smoked vs. snorted [49], but would hold peak brain levels of cocaine and dopamine constant in rats (while producing different rates of rise of both brain cocaine and dopamine levels [8], [16]).

The ability of a reward cue to support the learning of a new instrumental behaviour, in the absence of the primary reward, is a critical and rigorous test for the acquired incentive motivational power of the cue [41], [50]. In order to be able to dissociate conditioned reinforcement from primary reinforcement, we made it such that the operant response that led to presentations of the drug cues (lever-pressing) was distinct from that which previously led to the drug (nose-poking; [42]). Using this procedure, we found that cues which have been explicitly paired with self-administration of either rapid or slower i.v. cocaine are attributed with equivalent incentive motivational properties. This lack of effect of the speed of cocaine delivery was observed in two separate experiments using independent cohorts of subjects and different cue and cocaine self-administration parameters. These initial findings suggest that the greater addictive potential of rapidly delivered cocaine (e.g., smoked or injected cocaine relative to intranasal cocaine) occurs through mechanisms that might not involve differences in the ability of drug-paired cues to control behaviour.

It has been shown previously that operant responding for a cocaine-paired cue can persist for a long time (>2 months), in the absence of further pairings of the cue with drug [42]. In the current study, the ability to discriminate between the lever that produced the cocaine cue and an inactive lever that did not, abated with time. Indeed, one month following the cessation of cocaine self-administration, no lever discrimination was apparent, in either the 5- or the 90-s group. However, this was not due to ‘forgetting’ of the acquired rewarding value of the cue, because acute injections of amphetamine given following one month of withdrawal from cocaine selectively increased pressing on the lever that produced the cue. We do not know why lever discrimination dissipated with time and could only be seen following an amphetamine challenge. One possible explanation could be related to the fact that amphetamine and cocaine share common discriminative properties [51]. As such, amphetamine might have primed the performance of the operant response that led to presentations of the cocaine-paired cue by eliciting interoceptive signals similar to those elicited by cocaine. However, this is unlikely because the operant response that was reinforced by the cocaine cue (lever-pressing) was distinct from that which previously led to the delivery of cocaine (nose-poking). Thus, the lever-pressing response was never associated with the delivery of cocaine and its interoceptive effects. A parsimonious explanation for the effect of amphetamine is that compared to previous work showing persistent responding for drug cues [42], the drug cues in the current study acquired weaker incentive motivational properties, which were then enhanced by administration of amphetamine. Two factors that can influence the strength of a reward cue's reinforcing potency are the magnitude of the primary reward and the number of cue-reward pairings [52]. The number of cue-cocaine pairings earned is not given in the previous report [42], but the dose of cocaine that supported persistent responding for drug cues is slightly higher than ours (0.25 mg/infusion versus 0.5 mg/kg/infusion here, the latter being equivalent to 0.175 mg/infusion for a 350-g rat).

As mentioned above, the number of pairings between the conditioned stimulus and the primary reward can mediate the strength of conditioned reinforcement [52]. Therefore, in Experiment 2, we determined whether increasing the speed of cocaine delivery might reduce the number of cue-drug pairings necessary before the drug cues acquired incentive motivational value in their own right. We found that regardless of the speed of cocaine delivery, drug cues supported the establishment of a new instrumental response following twenty-four self-administration sessions (which provided 490 cue-drug pairings), but not following three self-administration sessions (which provided 30 cue-drug pairings). This suggests that regardless of the speed at which cocaine is delivered to the brain, extended exposure to cocaine and its associated environmental stimuli might be necessary before these stimuli are able to guide appetitive behaviour.

There are limitations to this study that should be considered when evaluating our conclusions. First, we only assessed one effect of drug cues on motivated behaviour—the ability to support the spontaneous learning of new actions. It must still be determined whether variations in the speed of self-administered cocaine injections influence other effects of drug cues that are relevant to addiction, including the ability to elicit approach, and invigorate or trigger drug-seeking behaviour. Interestingly, the same factors that influence how well a reward cue reinforces the learning of new behaviours [53] also influence how well drug cues are able to invigorate drug-taking and precipitate drug-seeking [54]. This suggests that all of these properties of a reward cue might have common substrates. As such, the present findings might predict that the speed of cocaine delivery would not influence the ability of drug cues to elicit approach or influence drug-seeking and drug-taking behaviour. This hypothesis remains to be evaluated. A second consideration is that we have assessed the effects of only one dose of cocaine. Cues paired with lower cocaine doses might acquire weaker incentive motivational properties, and increasing the speed of cocaine delivery might augment these properties. However, the level of operant responding for cocaine cues in the current study is quite similar to that seen in a report where both a lower and a higher dose of cocaine were tested (compare our Figure 3A to Figure 1 in [42]), and together our respective studies span the range of doses that reliably support the acquisition of cocaine self-administration behaviour in rats [55]. Finally, we have compared the effects of spatially isolated, environmental (i.e., exteroceptive) cues paired with rapid versus more sustained intravenous cocaine delivery. However, there are also potent *interoceptive* cues associated with cocaine intake, and the interoceptive signals produced by rapid versus sustained intravenous cocaine injections in our rats are likely not as different as the interoceptive signals produced by taking cocaine via different routes of administration in humans. Indeed, one can easily imagine that humans self-administering cocaine to the nasal mucosa versus intravenously or by inhalation would experience quite different sensations. It remains to be determined how the different peripheral signals associated with different routes of administration might acquire incentive motivational properties and contribute to drug-taking behaviour.

Cocaine can be taken by several different routes, including the intranasal, intravenous or inhalation routes. These routes differ in how much drug they deliver to the organism, but also in how fast [49], [56]. Cocaine taken by any of these routes can lead to addiction. However, it is generally agreed that addiction is more severe in subjects who inject or smoke the drug [1]–[7]. Compared to intranasal cocaine users, those who smoke or inject the drug take more of it, in higher doses, and for longer, report that they are less able to control their use and score higher on severity of addiction scales [1], [2]. Similar findings are reported for heroin [57]. Thus, drug addiction appears to be somehow qualitatively and/or quantitatively different in those who use rapid routes of drug delivery. If this is truly the case, then it is possible that this special population of addicts would benefit from tailored treatment approaches. At present, it is not known exactly what behavioural or neurobiological targets should form the basis of such customized treatment strategies. Our results show that the conditioned rewarding effects of drug cues are not different in rats that self-administer rapid vs. more sustained cocaine. As such, whatever behavioural and neurobiological mechanisms underlie the greater addictive of rapidly delivered cocaine, these are unlikely to include a greater sensitivity to drug cues. Although additional research is needed, the present findings suggest that interventions that diminish the responsiveness to drug cues might not be preferentially effective in reducing the pursuit and consumption of rapidly delivered cocaine.

## Supporting Information

Figure S1 **When in the presence of two novel levers, rats spontaneously press more on the lever that produces a conditioned reward (CR) than on the lever that does not (NCR).** Values are mean ± SEM. N = 6. *p<0.05 compared with NCR.
(EPS)
